# The Aryl Hydrocarbon Receptor (AhR) Is a Novel Gene Involved in Proper Physiological Functions of Pancreatic β-Cells

**DOI:** 10.3390/cells14010057

**Published:** 2025-01-06

**Authors:** Shuhd Bin Eshaq, Jalal Taneera, Shabana Anjum, Abdul Khader Mohammed, Mohammad H. Semreen, Karem H. Alzoubi, Mohamed Eladl, Yasser Bustanji, Eman Abu-Gharbieh, Waseem El-Huneidi

**Affiliations:** 1 Research Institute of Medical and Health Sciences, University of Sharjah, Sharjah P.O. Box 27272, United Arab Emirates; u21100624@sharjah.ac.ae (S.B.E.); jtaneera@sharjah.ac.ae (J.T.); anjum6repro@gmail.com (S.A.); amohammed@sharjah.ac.ae (A.K.M.); msemreen@sharjah.ac.ae (M.H.S.); ybustanji@sharjah.ac.ae (Y.B.); eabugharbieh@sharjah.ac.ae (E.A.-G.); 2 College of Medicine, University of Sharjah, Sharjah P.O. Box 27272, United Arab Emirates; meladl@sharjah.ac.ae; 3 College of Pharmacy, University of Sharjah, Sharjah P.O. Box 27272, United Arab Emirates; 4 Faculty of Pharmacy, Jordan University of Science and Technology, Irbid 22110, Jordan; 5 School of Pharmacy, The University of Jordan, Amman 11942, Jordan

**Keywords:** aryl hydrocarbon receptor (AHR), insulin secretion, glucose uptake, INS-1 cells, human islets

## Abstract

The Kynurenine pathway is crucial in metabolizing dietary tryptophan into bioactive compounds known as kynurenines, which have been linked to glucose homeostasis. The aryl hydrocarbon receptor (AhR) has recently emerged as the endogenous receptor for the kynurenine metabolite, kynurenic acid (KYNA). However, the specific role of AhR in pancreatic β-cells remains largely unexplored. This study aimed to investigate the expression of AhR in human pancreatic islets using publicly available RNA-sequencing (RNA-seq) databases and to explore its correlations with various metabolic parameters and key β-cell markers. Additionally, functional experiments were conducted in INS-1 cells, a rat β-cell line, to elucidate the role of Ahr in β-cell biology. RNA-seq data analysis confirmed the expression of AHR in human islets, with elevated levels observed in pancreatic islets obtained from diabetic and obese donors compared to non-diabetic or lean donors. Furthermore, AHR expression showed an inverse correlation with the expression of key β-cell functional genes, including insulin, PDX-1, MAFA, KCNJ11, and GCK. Silencing Ahr expression using siRNA in INS-1 cells decreased insulin secretion, insulin content, and glucose uptake efficiency, while cell viability, apoptosis rate, and reactive oxygen species (ROS) production remained unaffected. Moreover, Ahr silencing led to the downregulation of major β-cell regulator genes, Ins1, Ins2, Pdx-1, and Glut2, at both the mRNA and protein levels. In summary, this study provides novel insights into the role of AhR in maintaining proper β-cell function. These findings suggest that AhR could be a potential target for future therapeutic strategies in treating type 2 diabetes (T2D).

## 1. Introduction

The Kynurenine pathway is an essential biological pathway responsible for most tryptophan breakdowns in mammalian cells, generating various bioactive metabolites known as kynurenines. Among these metabolites, kynurenine (KYN) is metabolized by indole 2,3-dioxygenase (IDO) and tryptophan 2,3-dioxygenase (TDO) to produce kynurenic acid (KYNA) and quinolinic acid (QUIN), as well as 3-hydroxykynurenine (3-OH-KYN). These kynurenines have been implicated in modulating energy metabolism, the immune system, and neurotransmitter systems. Notably, KYNA has demonstrated neuroprotective effects in animal models [1,2].

Dysregulation of the kynurenine pathway has been linked to the development of Diabetes Mellitus (DM). Imbalances in kynurenine metabolites have been associated with chronic low-grade inflammation or stress observed in different types of DM [3]. In vitro studies have demonstrated that exposing human pancreatic islets to inflammatory cytokines can increase KYN and KYNA levels [4]. However, several cohort studies have reported reduced serum tryptophan, KYN, and KYNA levels in individuals with Type 1 Diabetes (T1D) [5,6]. The depletion in tryptophan could be due to the excessive oxidative stress in T1D that impairs the enzymatic activity in the kynurenine pathway, resulting in a reduction in KYN and KYNA production. On the other hand, most metabolomics studies have found a positive association between tryptophan, KYN, KYNA, and the development of T2 D [7,8], where higher levels of tryptophan increase the risk of T2D. Nevertheless, adopting a healthy lifestyle, including regular exercise, has been shown to promote the beneficial aspects of kynurenine metabolites [9]. These findings suggest a potential involvement of the kynurenine pathway in the pathogenesis of DM and highlight its significance in understanding the underlying mechanisms of the disease.

DiNatale et al. reported that HYNA is a potent AHR endogenous ligand that induces interleukin-6 (IL-6) [10]. AhR is a cytoplasmic transcription factor that belongs to the bHLH/PAS family of receptors. It is expressed in various human tissues, including the liver, lungs, pancreas, and heart, and regulates cellular processes such as stem cell maintenance, cell differentiation, immune modulation, and tumorigenesis [11]. AhR was initially recognized as a receptor for xenobiotic compounds, such as environmental toxins like 2,3,7,8-tetrachlorodibenzo-p-dioxin (TCDD) or natural compounds found in plants like polyphenols and flavonoids. Upon ligand stimulation, AhR translocates into the nucleus and forms a complex with a transcription factor called AhR nuclear translocator (ARNT). The activated AhR-ARNT complex recognizes specific DNA regions known as xenobiotic responsive elements (XREs) and initiates the transcription of genes, such as cytochrome P450 (CYP1A1) and CYP1B1 [12]. The link between DM and the endogenous receptor AhR has been studied in limited detail. Thackaberry et al. found that mice lacking AhR exhibited altered insulin regulation and responsiveness, suggesting its importance in insulin regulation [13]. However, the precise impact of AhR on β-cell function and its contribution to diabetes development or progression remains unclear. To better understand the physiological role of AhR in pancreatic β-cell function, we analyzed RNA-seqdata to investigate its expression profile in diabetic and control human pancreatic islets.

Additionally, we correlated AHR expression with HbA1c, BMI, age, and other key β-cell function genes. Furthermore, we performed in vitro functional studies using INS-1rat β-cells to elucidate the impact of AhR on pancreatic β-cell physiology. These investigations aim to shed light on the role of AhR in diabetes and enhance our understanding of its involvement in β-cell function.

## 2. Materials and Methods

### 2.1. RNA-Seq Expression Data

The publically available Gene Expression Omnibus (GEO) database was used to retrieve RNA-seq expression data from human pancreatic islets (GSE50398). As described, RNA-sequencing was performed using an Illumina’s TruSeq RNA Preparation Kit (Illumina, CA, USA). The output reads were aligned to the human reference genome (hg19) with STAR.17,18. Raw data were normalized using a trimmed mean of M-values and presented as fragments per kilobase of exon per million fragments mapped (FPKM) or transformed into log2 counts per million using the voom function (edgeR/limma R-packages) [14]. Pancreatic Islets were isolated from 72 donors with European ancestry; 21 were hyperglycemic/diabetic (10 females, 11 males, mean age of 61 years, mean of BMI 27.3, and HbA1c ≥ 6.5%) and 51 were normoglycemic/non-diabetic donors (33 males, 18 females, mean age of 55.6 years, mean of BMI 25.6 and HbA1c < 6%).

### 2.2. Cell Culturing and siRNA Transfection

As previously described, rat β-cells INS-1 (832/13) were cultured in RPMI medium supplemented with 10% FBS [15]. When cells reached ~60–70% confluency, they were transfected with 40 nM of two siRNA sequences targeting the Ahr gene (siRNA IDs: s130485 and s130486) or a negative control siRNA (Thermo Fisher Scientific, Waltham, MA, USA) using 1.0 µL of Lipofectamine^®^ 3000 in Opti-MEM^®^ Reduced-Serum medium (Thermo). The lipid–siRNA complex was then mixed with antibiotic-free RPMI. The prepared transfection media was added to the cells (500 µL per well) and incubated for 24 h at 37 °C. Twenty-four hours post-transfection, media was replaced with antibiotic-positive media and incubated overnight at 37 °C. Cells were then subjected to further molecular and metabolic assays.

### 2.3. RNA Extraction and Quantitative PCR (qPCR)

A standard RNA extraction kit, PureLink™ RNA Mini Kit (Invitrogen, Waltham, MA, USA; Cat No. 12183018A), was used for RNA extration. RNA concentration and quality were determined using NanoDrop 2000. The cDNA was synthesized using a high-capacity cDNA Reverse Transcription Kit (Thermo Fisher). Expression analysis was performed by SYBR-Green primers in a Quantstudio3-PCR system (Applied Biosystems, Waltham, MA, USA) (Table 1). The 2−ΔΔCt method was used to estimate the relative gene expression [16].

### 2.4. Insulin Secretion Assays

As previously described, the insulin secretion assay was conducted on transfected cells 48 h after transfection [15,16]. Cells were first washed and stabilized for 2 h in 1 mL secretion assay buffer (SAB) (pH 7.2, containing 114 mM NaCl, 4.7 mM KCl, 1.2 mM KH_2_PO_4_, 1.16 mM MgSO_4_, 20 mM HEPES, 2.5 mM CaCl_2_, 25.5 mM NaHCO_3_, and 0.2% Bovine Serum Albumin). Then, cells were incubated in 1 mL SAB supplemented with different glucose concentrations: either 2.8 mM, 16.7 mM, 2.8 mM plus 35 mM KCl, or 10 mM α-ketoisocaproic acid (α-KIC) for one hour. Afterwards, 500 µL of each conditioned SAB was collected and analyzed for insulin secretion using a rat insulin ELISA kit (Mercodia, Uppsala, Sweden, Catalog no. 10-1145-01). To measure insulin content, total protein was extracted from transfected cells using M-PER reagent (Thermo Fisher Scientific) and quantified with a Pierce BCA protein assay kit (Thermo Fisher Scientific). The protein was diluted at a 1:250 ratio and analyzed for insulin content with a rat insulin ELISA kit (Mercodia, Sweden). Insulin secretion and content were normalized to the total protein amount.

### 2.5. Glucose Uptake Assay

Transfected INS-1 cells were treated with a fluorescent glucose analog, 2-[N-(7-nitrobenz-2-oxa-1,3-diazol-4-yl) amino]-2-deoxy-glucose (2-NBDG) (Invitrogen #N13195), for 45 min at 37 °C [15,16]. Next, cell pellets were collected using trypsin, washed twice with cold PBS, and centrifuged at 1500 rpm for 5 min at 4 °C. Finally, the supernatant was discarded and the cells were suspended in 200 μL of cold PBS in each tube. Flow cytometry (BD FACS Aria™ III) was used to quantify and analyze cells at excitation 465 nm and emission at 540 nm wavelengths.

### 2.6. Intracellular Reactive Oxygen Species (ROS) Measurement

The ROS-Glo H_2_O_2_ assay was used to measure ROS production levels in transfected cells (Promega, Madison, WI, USA), as described previously [15,16].

### 2.7. Apoptosis and Cell Viability Assay

The annexin V-FITC/propidium iodide (PI) test for apoptosis and MTT assay for cell viability were assessed in transfected cells 48 h post-transfection, as previously described [15,16].

### 2.8. Western Blot Analysis

Western blot expression analysis was performed as previously described [15,16], with the following antibodies: GLUT2 (Anti-rabbit; 1:1000, #ab54460), GCK (Anti-rabbit; 1:1000; #ab37796), PDX1 (Anti-rabbit; 1:3000, #ab47267) (from Abcam, Cambridge, UK), Insulin (Anti-mouse; 1:1000; #8138s), INSRβ (Anti-rabbit; 1:1000; #E9L5V) (from Cell Signaling Technology, Danvers, MA, USA), and β-actin (1:5000; #A5441, Sigma-Aldrich, Burghausen, Germany). The secondary antibodies were anti-mouse #7076S and anti-rabbit #7074S from Cell Signaling Technology. Proteins were visualized using an ECL substrate kit (Bio-Rad, Hercules, CA, USA). Protein bands were analyzed using Bio-Rad Image Lab software (version 6.1, ChemiDocTM Touch Gel Western Blot Imaging System; Bio-Rad, Hercules, CA, USA), whereas protein band quantification was applied using Image J software. As an endogenous control, β-actin was used in all experiments.

### 2.9. Immunostaining

INS-1 cells were seeded on coverslips placed in 24-well cell culture plates. Then, the media was removed 48 h post-transfection and cells were washed with 1 mL PBS for 5 min. Next, cells were fixed using 500 μL of 4% paraformaldehyde. After 15 min, cells were washed thrice with PBS and then permeabilized with 500 μL of 0.2% Triton X-100. Subsequently, 500 μL of 2% fetal bovine serum in 1% Triton X-100 was used to block the cells for 1 h. After removal of the blocking solution, the primary antibody for AhR (#MA1-514, Invitrogen, Waltham, MA, USA) was prepared in PBS with 0.1% Triton X-100 (1:400) and incubated overnight at 4 °C with gentle agitation. The following day, cells were washed thrice for 5 min each with PBS, 0.1% Triton X-100. Then, cells were incubated with Fluorochrome 488-conjugated secondary antibody (anti-mouse) diluted in PBS, 0.1% Triton X-100 (1:1000) for 90 min in the dark at room temperature. Cells were then rinsed with PBS, 0.1% Triton X-100 and coverslips were mounted and analyzed using the confocal microscope (A1R Confocal Laser Microscope System, Nikon Inc., Tokyo, Japan). As a negative control, cells stained only with secondary antibodies were used as a non-specific binding control.

### 2.10. Statistical Analysis

The obtained data are illustrated by Standard Deviation (±SD) or Standard Error of Mean (±SEM). GraphPad Prism (version 8.0.0 for Windows, GraphPad Software, San Diego, CA, USA) was used to perform all statistical analyses. Student’s *t*-test and the nonparametric Mann–Whitney test were used to compare gene expression in diabetic versus non-diabetic islets. In addition, a nonparametric Spearman’s test was used to correlate gene expressions and other phenotypes. Differences were considered significant at *p* < 0.05.

## 3. Results

### 3.1. mRNA Expression of AHR in Human Pancreatic Islets

**Figure 1:**
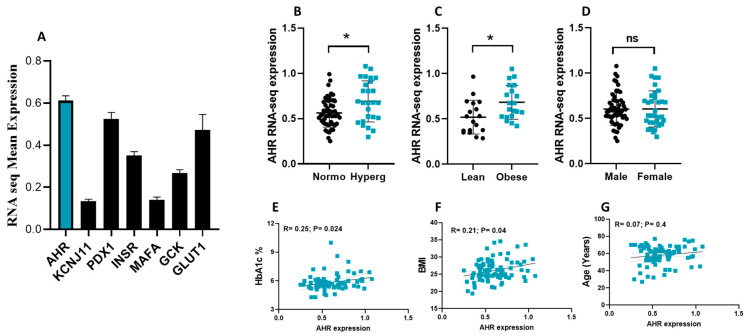
Expression analysis of AHR in human pancreatic islets. (**A**) RNA-seq expression of AHR, KCNJ11, PDX1, INSR, MAFA, GCK, and GLUT1 in human islets obtained from non-diabetic donors (n = 49). (**B**). AHR expression levels in human islets obtained from diabetic/hyperglycemic donors (n = 25) compared to nondiabetic/normoglycemic islet (n = 49) donors. (**C**) AHR expression levels in human islets obtained from lean donors (n = 18; BMI below 24) compared to obese donors (n = 19; BMI above 29). (**D**) AHR expression levels in human islets obtained from male donors (n = 53) compared to female donors (n = 33). Correlation of AHR expression with HbA1c% (n = 66) (**E**), BMI (n = 87) (**F**), or age (n = 87) (**G**). *; *p* > 0.05, ns; not significant. Bars represent mean ± SEM. Nonparametric Mann–Whitney *t*-tests were used in (**B**–**D**). Nonparametric Spearman’s correlation test was used in (**E**–**G**).

The expression pattern of AHR in pancreatic islets of both diabetic and non-diabetic individuals was profiled using publicly available RNA-seq data. Figure 1A illustrates that AHR is expressed in human pancreatic islets at levels comparable to the β-cell functional gene GLUT1 and PDX-1 but higher than KCNJ11, INSR, MAFA, or GCK. Further analysis demonstrated a significant increase (*p* < 0.05) in AHR expression in diabetic/hyperglycemic or obese donors compared to non-diabetic/normoglycemic or lean donors (Figure 1B,C). Moreover, no differences were observed in AHR expression between pancreatic islets obtained from male or female donors (Figure 1D). Interestingly, the expression of AHR showed a positive correlation with HbA1c levels and BMI (*p* < 0.05) (Figure 1E,F), indicating a potential association between AHR and metabolic parameters. However, no significant correlation was observed between AHR expression and age (Figure 1G).

**Figure 2:**
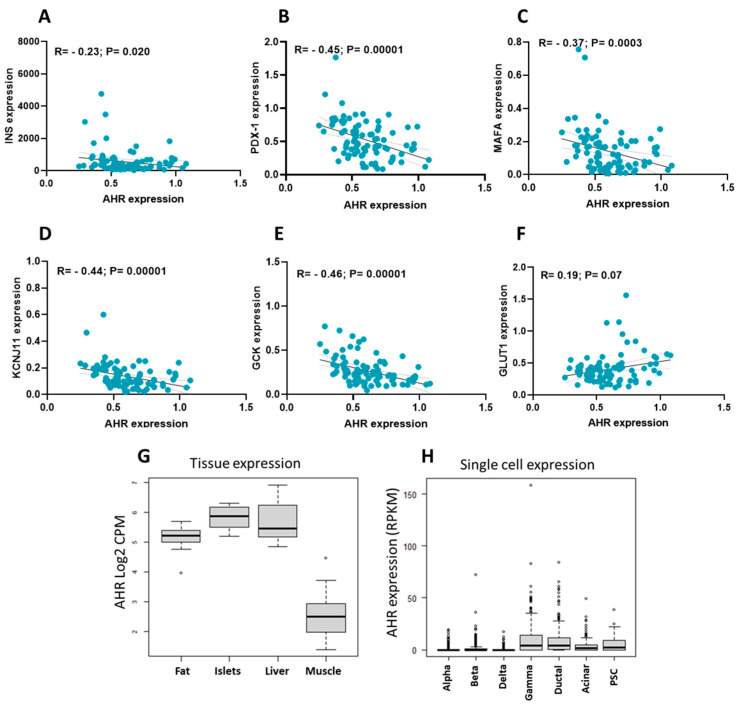
Expression correlations of AHR with key pancreatic β-cell markers. Expression of AHR was correlated with INS (**A**), PDX1 (**B**), MAFA (**C**), KCNJ11 (**D**), GCK (**E**), and GLUT1 (**F**) using nonparametric Spearman’s correlation. (**G**) Expression levels of AHR in human fat tissue (n = 12), pancreatic islets (n = 12), liver (n = 12), and skeletal muscle tissues (n = 12), obtained from the same donors. (**H**) Expression levels of AHR in sorted pancreatic cells, ductal, acinar, or PSC as obtained from Islet Gene View (IGV) web tool.

Co-expression analysis was performed with essential β-cell functional markers to further explore the functional implications of AHR expression. The results revealed a negative correlation between AHR and INS, PDX-1, MAFA, KCNJ11, and GCK (Figure 2A–E), suggesting a potential regulatory relationship between AHR and these key β-cell markers. Surprisingly, no significant correlation was found between AHR expression and the GLUT1 gene (Figure 2F).

Finally, to gain a broader understanding of AHR expression across different metabolic tissues, the Islet Gene View (IGV) web tool (https://mae.crc.med.lu.se/IsletGeneView/) (accessed on 12 June 2024) was utilized. The results in Figure 2G demonstrate that AHR exhibited higher expression levels in human islets, liver, and fat than in the muscle tissues. Additionally, the data from IGV revealed that AHR was expressed in sorted pancreatic β-cells, other islet cells, and pancreatic cell types (Figure 2H), suggesting its potential involvement in various pancreatic functions.

Overall, these findings shed light on the relationship between AHR expression, metabolic parameters, β-cell functional markers, and expression patterns across different metabolic tissues and pancreatic cell types, contributing to our understanding of the potential role of AHR in metabolic regulation.

### 3.2. siRNA Silencing of Ahr in INS 1 Impairs Insulin Secretion and Glucose Uptake

**Figure 3:**
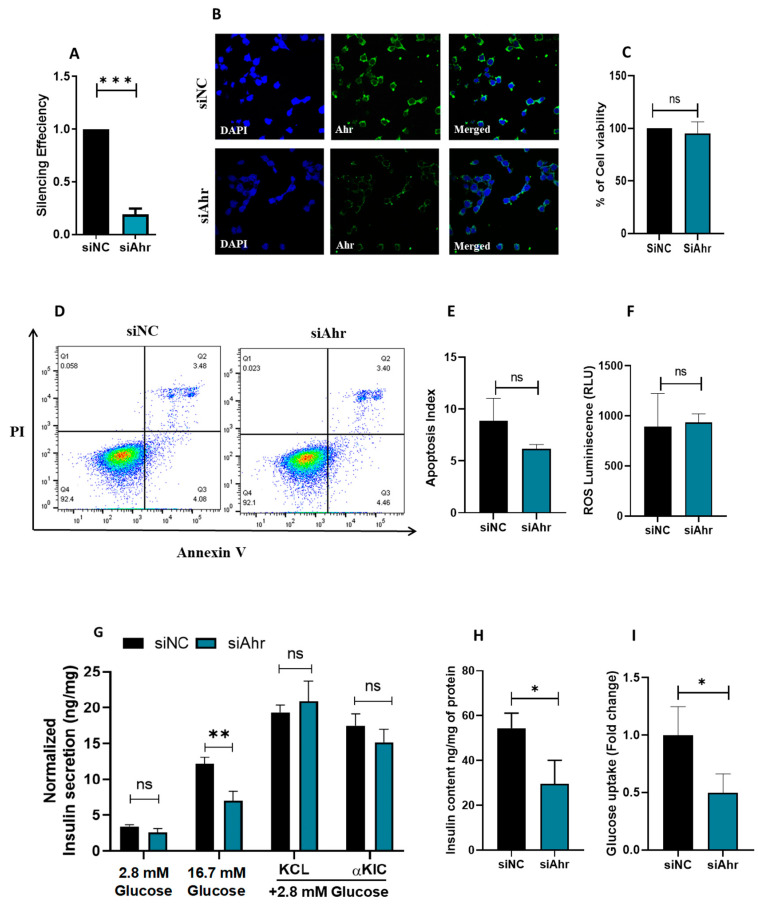
Silencing of Ahr and its impact on INS-1 cell function. (**A**) Analysis of mRNA expression of AhR 48 h after siRNA transfection as determined by qPCR in Ahr-silenced or control cells. (**B**) Confocal images of immunofluorescent staining of AHR protein in INS-1 with or without Ahr silencing. Blue is DAPI nuclear staining and green is AHR protein staining. The overlay of two markers is shown in the merged image. Magnification 60× (n. of experiments = 1). (**C**) Cell viability assay using MTT test. (**D**,**E**) Apoptosis analyzed by flow cytometry analysis using Annexin V-PI staining (**D**). The left panel denotes a summary of the apoptosis results (**E**). (**F**) ROS production measurements determined by luminescence-based analysis. (**G**) Normalized insulin secretion was stimulated in control or Ahr-silenced cells at 2.8 mM glucose, 16.7 mM glucose, and 2.8 mM glucose with KCL or αKIC. (**H**) Insulin content measurements relative to the total protein concentration. (**I**) Glucose uptake efficiency evaluated by flow cytometry. Data were acquired from three independent experiments unless otherwise mentioned. Bars display the mean ± SD. * *p* < 0.05, ** *p* < 0.01, *** *p* < 0.001, ns; not significant.

To elucidate the functional significance of Ahr in pancreatic β-cells, we employed siRNA to abolish Ahr expression in INS-1 cells. As shown in Figure 3A, Ahr mRNA expression was substantially reduced by 80% compared to the negative control (*p* < 0.05) 48 h after transfection. This was also validated on protein levels, as depicted by immunostaining (Figure 3B). Importantly, the silencing of Ahr had no notable impact on cell viability, as demonstrated by the MTT assay (Figure 3C), apoptosis rate as assessed by Annexin-V-PI staining (Figure 3D,E), or reactive oxygen species (ROS) production (Figure 3F).

To assess the impact of Ahr silencing on insulin secretion, transfected INS-1 cells were stimulated with glucose concentrations of 2.8 mM or 16.7 mM for one hour. The results revealed a significant decrease in glucose-stimulated insulin secretion (GSIS) in Ahr-silenced cells compared to controls at both 2.8 mM glucose (25%; *p* < 0.05) and 16.7 mM glucose (50%; *p* < 0.05) (Figure 3G). However, cells stimulated with 2.8 mM glucose along with either KCL (a depolarizing agent) or KIC (a stimulator of insulin secretion via mitochondrial metabolism and ATP production) revealed no notable differences observed between Ahr-silenced cells and control cells (Figure 3G). However, insulin content measurements in Ahr-silenced cells showed a significant decrease compared to control cells (*p* < 0.05), indicating a potential role of AhR in regulating insulin content (Figure 3H). Additionally, we explored the effects of Ahr silencing on glucose uptake rate. The results demonstrated a significant decrease of 50% in glucose uptake in Ahr-silenced cells compared to the control (*p* < 0.05) (Figure 3I). These results highlight the possible significance of Ahr in controlling essential aspects of glucose metabolism and pancreatic β-cell activity.

### 3.3. AhR Silencing Affects the Expression of β-Cell Functional Genes

**Figure 4:**
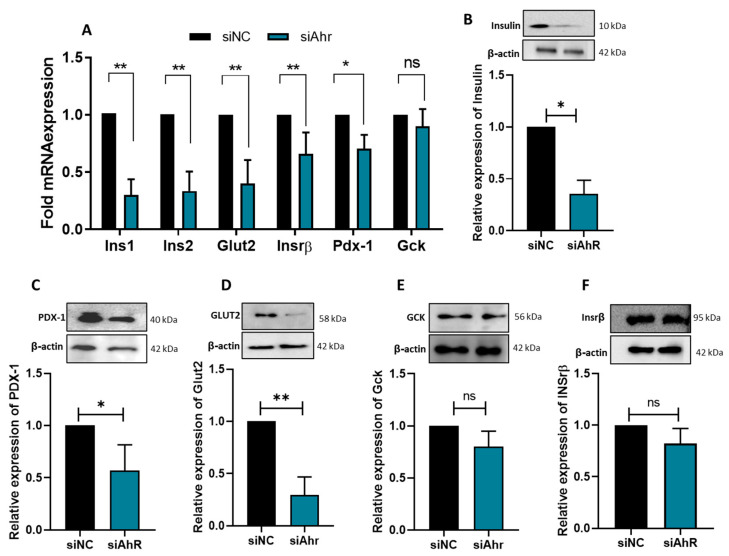
Impact of Ahr silencing on key β-cell function genes. (**A**) mRNA expression of Ins1, Ins2, Glut2, Insrβ, Pdx-1, and Gck in Ahr-silenced cells compared to control cells. Protein expression of (**B**) Pro/Insulin, (**C**) PDX-1, (**D**) GLUT2, (**E**) GCK, and (**F**) INSRβ relative to β-actin endogenous protein. Bars indicate mean ± SD fold changes in protein expression from three independent experiments. * *p* < 0.05, ** *p* < 0.01, ns; not significant.

Next, we aimed to investigate how Ahr silencing affected the mRNA and protein expression of crucial β-cell functional genes. The mRNA expression of Ins1, Ins2, Pdx-1, Glut2, and Insrβ was significantly (*p* < 0.05) lower in Ahr-silenced cells than in control cells (Figure 4A). In contrast, the expression levels of Gck showed a nonsignificant alteration (Figure 4A). These findings were further validated using Western blot analysis, which demonstrated significant decreases in the protein expression of Pro/Insulin (~65%; *p* < 0.05) (Figure 4B), PDX-1 (~45%; *p* < 0.05) (Figure 4C), and GLUT2 (~75%; *p* < 0.05) (Figure 4D) in Ahr-silenced cells. Conversely, INSRβ and GCK expression levels remained unchanged (Figure 4E,F). These findings add to our knowledge of the molecular mechanisms behind AhR’s function in β-cell biology by demonstrating that it is essential for controlling insulin synthesis and glucose sensing in β-cells.

## 4. Discussion

The findings of this study have demonstrated that normal β-cell functions were impacted in a number of ways by the silencing of AhR in INS-1 cells. This includes decreased glucose uptake efficiency, decreased insulin production, and downregulated levels of Ins1, Ins2, Glut2, and Pdx-1 expression without compromising cell survival or triggering apoptosis. According to the functional data obtained, AhR is an essential regulator of pancreatic β-cell function.

To date, various studies revealed a role of AhR in regulating multiple cellular activities [17,18,19,20,21,22]. For instance, an earlier study illustrated that hyperactivity of AhR may allow tumor survival and escape immune surveillance by activating pro-inflammatory signaling and establishing an immune-suppressive tumor microenvironment [17]. However, our study is the first to link AhR activity with pancreatic β-cells. This indicates that the activity of AhR is cell-type-dependent and/or affected by the surrounding microenvironment.

Moreover, the activity of the AHR protein has previously been correlated with energy metabolism and the development of metabolic disorders such as obesity and insulin resistance [23,24,25]. For example, one study found that the mRNA expression of Ahr is upregulated in peripheral blood mononuclear samples (PBMCs) collected from metabolically healthy obese and T2D patients, and that this upregulation is positively linked to insulin resistance and hyperinsulinemia [26]. Consistent with these findings, our study revealed an upregulation of AHR mRNA expression in islets obtained from donors with diabetes and obesity (Figure 1).

The observed positive correlation between AHR gene expression and HbA1c levels, along with BMI, coupled with the negative correlation with essential β-cell genes such as INS, PDX-1, MAFA, and KCNJ11, strongly suggests a pivotal role of AHR in the pathophysiology of T2D. These correlations indicate that increased AHR expression may be associated with impaired β-cell function and insulin secretion. Specifically, the downregulation of key regulatory genes like PDX-1 and MAFA, which are crucial for maintaining insulin synthesis and β-cell identity, implies that heightened AHR activity might contribute to the dysfunction of pancreatic β-cells. This dysregulation may lead to decreased insulin production and glucose homeostasis, thereby exacerbating the metabolic disturbances characteristic of T2D. Overall, these findings underscore the potential of AHR as a critical player in the development and progression of T2D.

The elevated expression of AHR in islets from diabetic or hyperglycemic donors presents a paradox, as Ahr silencing is associated with reduced insulin secretion in INS-1 cells. This discrepancy may be due to the complex interplay of co-expression relationships among various genes, which can be influenced by factors such as medication, genetics, local environmental conditions, and metabolic heterogeneity (including glucotoxicity, lipotoxicity, hyperinsulinemia, and insulin resistance) [27]. Additionally, the silencing of Ahr may activate compensatory mechanisms to preserve cellular homeostasis. These mechanisms could inadvertently down-regulate the expression of critical genes like Pdx1 and NeuroD1, which may be interconnected within the same regulatory network or pathway. Another potential explanation involves feedback loops; the expression of one gene can significantly influence the expression of others. Silencing Ahr might disrupt these feedback loops, resulting in unexpected alterations in gene expression.

Furthermore, this study has shown that the silencing of AhR impaired glucose-stimulated insulin secretion in INS-1 cells at basal and stimulation levels. However, this impairment was not changed when cells were stimulated with glucose along with KCL or αKIC. Importantly, insulin secretion impairment was associated with reduced insulin content. This indicates that the effect of AhR-silencing in reducing insulin secretion is more related to defects in insulin biosynthesis or glucose sensing rather than affecting insulin exocytosis and mitochondrial metabolism.

In line with this postulation, the data showed that the level of insulin mRNA and protein expression, as well as insulin gene regulator PDX-1 [28,29], was significantly reduced in AhR-silenced cells compared to control cells (Figure 3), indicating that AHR has a functional role in regulating the insulin production pathway. Likewise, a preceding in vivo study demonstrated that female mice lacking AhR expression had reduced insulin levels in their plasma and unbalanced glucose homeostasis [13]. However, contradictory results have been reported regarding the influence of AHR on glucose homeostasis and insulin secretion. For example, a study has shown that subjecting human islets to AHR exogenous ligands such as TCDD impaired insulin secretion and promoted apoptosis [30]. Such differences in the effect of AHR activity on insulin regulation indicate the complexity of AHR activity in metabolic syndrome and how it acts in both a context- and ligand-specific manner.

Furthermore, Ahr-silenced cells had much lower glucose uptake, as evidenced by the decreased expression of the Glut2 transporter and Gck at both the mRNA and protein levels. Pancreatic β-cells rely on GLUT2 and GCK as their principal glucose transporters and sensors [31,32,33,34]. Therefore, glucose sensing or uptake impairment will lead to elevated blood glucose levels and increase the incidence of T2D [35]. Similarly, previous studies have shown that deleting the Ahr partner protein ARNT in β-cells may affect Glut2 and Gck expression [36,37].

A previous study found that AHR exogenous ligands such as TCDD lead to downregulated expression of INSRβ in adipocyte tissue by decreasing the expression of proteins involved in the insulin signaling pathway through Tumor Necrosis Factor-α (TNF-α) production [38]. However, AhR downregulation by siRNA showed no effect on the levels of Insrβ. Aligned with these data, Ahr-silenced INS-1 cells exhibited an insignificant change in the protein level of INSRβ compared to the control cells. This indicates that AhR alone cannot affect insulin receptor activity unless exogenous ligands activate it as environmental toxins. Moreover, it is known that AhR modulates cell proliferation and apoptosis among various cell types such as cancer and immune cells [39]; our results revealed that Ahr silencing does not affect cell viability, nor the induction of apoptosis. The discrepancies can be attributed to several factors related to experimental design and methodology. In the first study, AhR knockout (AhR−/−) mice demonstrated increased apoptosis in the context of experimental autoimmune uveitis (EAU), as evidenced by TUNEL staining, which indicated a higher number of apoptotic cells compared to wild-type mice. In the second study, TCDD was used to induce the CYP1A enzyme, which resulted in a pronounced increase in mouse beta cell death.

Additionally, it has been shown that AHR overstimulation by exogenous ligands as pollutants was associated with an elevation in the generated ROS [40]. Other studies showed that the binding of AhR with other ligands such as flavonoids activates antioxidant enzymes to protect against oxidative stress [41]. This study found no difference in ROS levels in Ahr-silenced cells compared to the control cells. This contradiction may stem from the different activation pathways of AhR in acute versus chronic inflammatory conditions, resulting in distinct cellular responses. Additionally, AhR signaling is known to be influenced by the type of ligand present, particularly the presence of endogenous ligands, which can further modulate its effects. These differences highlight the complexity of AhR signaling and its influence on various cellular processes, suggesting that AhR activity is cell-type-specific and shaped by the surrounding microenvironment. 

## 5. Conclusions

Our findings indicate that AHR is crucial in regulating pancreatic β-cell activity. Further research is necessary to explore the underlying mechanisms that govern AHR expression. This will provide valuable insights into its significance in pancreatic β-cell biology and its potential implications in diverse physiological and pathological processes. Exploring the effect of both agonist and antagonist beta cells’ functions will be an important future strategy to deepen our understanding of the receptor activity. Additionally complementing the conducted knockdown experiments with overexpression studies will further validate and refine our findings. Finally, using an in vivo model would provide more biologically relevant insights into AhR function by looking at tissues and cell types impaired in diabetes and metabolic syndromes, such as muscles, liver, and adipocytes.

## Figures and Tables

**Table 1:** PCR primer sequences.

Gene	Forward (5’-3’)	Reverse (5’-3’)
*Hprt*	TTGTGTCATCAGCGAAAGTGG	CACAGGACTAGAACGTCTGCT
*Insr*	GAGTCCAGAGCTCACCAAGG	TCTCAGACTCCACACAACGC
*Gck*	CCTGGGAGGAACCAACTTCA	TCTTGTGCTTCATCATCTCGGC
*Pdx1*	TTCAGTGCTAATCCCCCTGC	ACTTCCCTGTTCCAGCGTTC
*Glut2*	GCAACATGTCAGAAGACAAGATCA	TGTCATATCCGAACTGGAAGGA
*Ahr*	GCAAGGTAAGGGCTTTCTACAG	GTCTGAAGGTGGCCAATGCT

## Data Availability

Data will be available from the corresponding author upon request.

## Abbreviations

Hypoxanthine-guanine phosphoribosyl transferase (Hprt), insulin receptor (InsR), glucokinase (Gck), pancreatic and duodenal homeobox 1 (Pdx1), glucose transporter2 (Glut2), aryl hydrocarbon receptor (Ahr), MAF BZIP transcription factor A (MAFA), potassium inwardly rectifying channel subfamily J member 11 (KCNJ11), body mass index (BMI), glucose transporter1 (GLUT1), glycated hemoglobin (HbA1c).
